# Supplementary material for: The Immunological Landscape of M1 and M2 Macrophages and Their Spatial Distribution in Patients with Malignant Pleural Mesothelioma
Source: Cancers (Basel). 2023 Oct 24;15(21):5116. doi: 10.3390/cancers15215116 (PMC10650059; doi:10.3390/cancers15215116)
Supplement: Supplementary file 1 [file cancers-15-05116-s001.zip › cancers-2625425-supplementary.pdf]

Supplementary Table S1. Frequency of demographic and clinicopathologic characteristics of the patients in the TCGA-MESO database as a computational exploratory cohort (n=87).

Characteristic	No.	%
**Age** (range, 28-81 y)		
≤64 y	45	51.7
>64 y	42	48.3
**Sex**		
Male	71	81.6
Female	16	18.4
**Histological subtype**		
Epithelioid	62	71.3
Biphasic	23	26.4
Sarcomatoid	2	2.3
**T category**		
T1	14	16.1
T2	26	29.9
T3	32	36.8
T4	13	14.9
TX	2	2.3
**N category**		
N0	44	50.6
N1	10	11.5
N2	26	29.9
N3	3	3.4
NX	4	4.6
**M category**		
M0	57	65.5
M1	3	3.4
MX	27	31.0
**Vital status at last follow-up**		
Alive	13	14.9
Dead	74	85.1

Supplementary Table S2. Antibody optimization by multiplex immunofluorescence using the Opal fluorophores (Akoya Biosciences).

Antibody	Clone	Vendor	Catalog #	AR	Ab. dilution	F	F dilution
CK	AE1/AE3	Dako	M351501-2	pH6	1:25	540	1:100
CD68	PG-M1	Dako	M0876	pH9	1:25	520	1:100
CD206	Polyclonal	Invitrogen	PA5-83759	pH6	1:100	570	1:100
CD86	E2G8P	Cell Signaling	91882S	pH9	1:100	620	1:100
CD163	10D6	Leica	NCL-L-CD163	pH9	1:100	690	1:200
Arginase-1	D4E3M™	Cell Signaling	93668S	pH9	1:200	650	1:100
PD-L1	E1L3N	Cell Signaling	13684S	pH6	1:100	480	1:100
MRP8+MRP14	S100A8/A9	OriGene	BM4025	pH6	1:50	780D/R	1:100/1:25

CK, cytokeratins; AR, antigen retrieval; Ab, antibody dilution; F, fluorophore.

The Opal Polaris 780 Fluorophore is a two parts reaction and contains opal TSA-DIG (1:100) and Opal Polaris 780 (1:25)

Supplementary Table S3. Possible macrophage phenotypes found with the multiplex immunofluorescence panel according to positive and negative expression of markers.

Phenotype	Marker
Total malignant cells	CK+
Total macrophages	CD68+
M1	CD68+MRP8-14+CD163^{neg}CD206^{neg}Arg-1^{neg}
M1	CD68+CD86+CD163^{neg}CD206^{neg}Arg-1^{neg}
M1	CD68+CD86+MRP8-14+CD163^{neg}CD206^{neg}Arg-1^{neg}
M2	CD68+CD163+MRP8-14^{neg}CD86^{neg}
M2	CD68+CD206+MRP8-14^{neg}CD86^{neg}
M2	CD68+CD163+CD206+MRP8-14^{neg}CD86^{neg}
M2a	CD68+CD163+Arg-1+MRP8-14^{neg}CD86^{neg}
M2a	CD68+CD206+Arg-1+MRP8-14^{neg}CD86^{neg}
M2a	CD68+CD163+CD206+Arg-1+MRP8-14^{neg}CD86^{neg}
M2b	CD68+CD86+MRP8-14^{neg}
M2c	CD68+CD163+Arg-1^{neg}MRP8-14^{neg}CD86^{neg}
M2c	CD68+CD206+Arg-1^{neg}MRP8-14^{neg}CD86^{neg}
M2c	CD68+CD163+CD206+Arg-1^{neg}MRP8-14^{neg}CD86^{neg}
Malignant cells expressing PD-L1	CK+PD-L1+
Macrophages expressing PD-L1	CD68+PD-L1+
M1 expressing PD-L1	CD68+MRP8-14+PD-L1+CD163^{neg}CD206^{neg}Arg-1^{neg}
M1 expressing PD-L1	CD68+CD86+ PD-L1+CD163^{neg}CD206^{neg}Arg-1^{neg}
M1 expressing PD-L1	CD68+CD86+MRP8-14+PD-L1+CD163^{neg}CD206^{neg}Arg-1^{neg}
M2 expressing PD-L1	CD68+CD163+PD-L1+MRP8-14^{neg}CD86^{neg}
M2 expressing PD-L1	CD68+CD206+ PD-L1+MRP8-14^{neg}CD86^{neg}
M2 expressing PD-L1	CD68+CD163+CD206+PD-L1+MRP8-14^{neg}CD86^{neg}
M2a expressing PD-L1	CD68+CD163+Arg-1+PD-L1+MRP8-14^{neg}CD86^{neg}
M2a expressing PD-L1	CD68+CD206+Arg-1+PD-L1+MRP8-14^{neg}CD86^{neg}
M2a expressing PD-L1	CD68+CD163+CD206+Arg-1+PD-L1+MRP8-14^{neg}CD86^{neg}
M2b expressing PD-L1	CD68+CD86+PD-L1+MRP8-14^{neg}
M2c expressing PD-L1	CD68+CD163+PD-L1+Arg-1^{neg}MRP8-14^{neg}CD86^{neg}
M2c expressing PD-L1	CD68+CD206+PD-L1+Arg-1^{neg}MRP8-14^{neg}CD86^{neg}
M2c expressing PD-L1	CD68+CD163+CD206+PD-L1+Arg-1^{neg}MRP8-14^{neg}CD86^{neg}

M, macrophage; neg, negative.

Supplementary Table S4. Functional enrichment of reactome pathways.

Description	Identifier	Strength
Metal sequestration by antimicrobial proteins	HSA-6799990	2.56
CD163 mediating an anti-inflammatory response	HSA-9662834	2.38
Interleukin-10 signaling	HSA-6783783	2.08
Regulation of TLR by endogenous ligand	HSA-5686938	2.06
Interleukin-4 and interleukin-13 signaling	HSA-6785807	1.78
Signaling by interleukins	HSA-449147	1.24
Neutrophil degranulation	HSA-6798695	1.06
Immune system	HSA-168256	0.89
Innate immune system	HSA-168249	0.87

Supplementary Table S5. Cox proportional hazards regression model of overall survival in patients with malignant pleural mesothelioma comparing low with high densities of different macrophage phenotypes, adjusted for histology type and asbestos exposure.

Variable	B	SE	Wald	HR	95% CI for Exp(B)	*P**
Histologic type (Epithelioid vs non-epithelioid)	-0.422	0.517	0.668	.656	0.238-1.805	0.414
Asbestos exposure (Yes vs. no)	-0.168	0.467	0.130	.845	0.338-2.111	0.719
Low vs. high densities						
Total CD68+	0.199	0.963	0.043	1.221	0.185-8.052	0.836
(M1) CD68+MRP8-14+CD163^{neg}CD206^{neg}Arg-1^{neg}	-0.458	0.434	1.114	.633	0.270-1.481	0.291
(M1) CD68+CD86+CD163^{neg}CD206^{neg}Arg-1^{neg}	-0.283	0.675	0.175	.754	0.201-2.832	0.676
(M1) CD68+CD86+MRP8-14+CD163^{neg}CD206^{neg}Arg-1^{neg}	-2.832	1.652	2.937	.059	0.002-1.502	0.087
(M2) CD68+CD163+MRP8-14^{neg}CD86^{neg}	1.032	1.308	0.623	2.807	0.216-36.434	0.430
(M2) CD68+CD206+MRP8-14^{neg}CD86^{neg}	-1.953	0.913	4.582	.142	0.024-0.848	**0.032**
(M2) CD68+CD163+CD206+MRP8-14^{neg}CD86^{neg}	1.860	0.790	5.542	6.422	1.365-30.205	**0.019**
(M2a) CD68+CD163+Arg-1+MRP8-14^{neg}CD86^{neg}	0.437	0.554	0.624	1.549	0.523-4.586	0.430
(M2a) CD68+CD206+Arg-1+MRP8-14^{neg}CD86^{neg}	-0.917	0.744	1.520	.400	0.093-1.718	0.218
(M2a) CD68+CD163+CD206+Arg-1+MRP8-14^{neg}CD86^{neg}	0.315	0.602	0.274	1.371	0.421-4.464	0.601
(M2b) CD68+CD86+MRP8-14^{neg}	-0.224	0.622	0.130	.799	0.236-2.703	0.718
(M2c) CD68+CD163+Arg-1^{neg}MRP8-14^{neg}CD86^{neg}	-0.441	1.292	0.117	.644	0.051-8.089	0.733
(M2c) CD68+CD206+Arg-1^{neg}MRP8-14^{neg}CD86^{neg}	-0.261	0.660	0.157	.770	0.211-2.810	0.692
(M2c) CD68+CD163+CD206+Arg-1^{neg}MRP8-14^{neg}CD86^{neg}	1.268	0.850	2.228	3.555	0.672-18.802	0.135

Note: B, unstandardized regression weight; SE, multiple linear regression; Wald, Wald test; HR, hazard ratio; CI, confidence interval; (M) macrophages; neg, negative.
*Boldface indicates a statistically significant difference using Cox proportional hazards model with P-values unadjusted for clinicopathologic variables. The table shows the analysis of densities of the cell phenotypes adjusted for clinicopathologic features.

Supplementary Figure S1. Schematic of the action points associated with tumor-associated macrophages targeted by immunotherapy investigated in clinical trials. The schematic represents the most relevant pathways targeted by drugs investigated in clinical trials. Note the wide range of agents with action on different cell components, from targets such as immune checkpoint inhibitors to targets of direct action on DNA transcription, such as action on histones. The images were generated using BioRender (https://www.biorender.com/).

Supplementary Figure S2. Representative examples of unmixed and mixed multispectral images from controls of human reactive tonsil and tuberculous **pneumonitis.** Unmixed images (20× magnification; scale bars represent 50 µm on each image) showing the individual marker's expression plus DAPI (4′,6-diamidino-2-phenylindole) and their composite mixed image contained all the markers plus DAPI from human reactive tonsil and acute tuberculous pneumonia: cytokeratin (CK), CD68, CD206, CD86, CD163, Arg-1, MRP8-14, and PD-L1. The

images were generated using the PhenoImager 1.0.13 scanner system and inForm 2.4.8 image analysis software (Akoya Biosciences).

Supplementary Figure S3. Tumor gene expression according to sex. Boxplots showing a significant correlation between high expression of transcripts per million of *CD274, IL12B*, and *INHBA* and male patients (*P*=0.0004, *P*=0.001, and *P*=0.006, respectively). Data from 87 samples was used. Boxplots show the median (bar), interquartile range values (Q2 and Q3), and maximum and minimal values. The data were downloaded from UALCAN, from the expression profiles for each gene analyzed. Expression and group status were compared by the platform itself using Student t-test. **P*<0.05; ***P*<0.01.

Supplementary Figure S4. Tumor gene expression according to histotype. Boxplots showing high expression of *IL1B* and *INHBA* in the biphasic histotype compared to epithelioid (*P*=0.03, *P*=0.05, respectively), while epithelioid showed upregulation of *CHI3L1* compared to biphasic (*P*=0.02). Furthermore, *IL6* showed high expression in the biphasic histotype compared to diffuse malignant (*P*=0.03). Lastly, *CHI3L1* showed high expression in diffuse malignant compared to biphasic (*P*=0.006). Data from 87 samples were used. Boxplots show the median (bar), interquartile range values (Q2 and Q3), and maximum and minimal values. The data were downloaded from UALCAN, from the expression profiles for each gene analyzed. Expression and group status were compared by the platform itself using Student t-test. **P*<0.05; ***P*<0.01.

Supplementary Figure S5. Tumor gene expression according to clinical stage. Boxplots showing significant overexpression of *CD163* and *MRC1* in stage IV compared to stage II (*P*=0.04, *P*=0.02, respectively), and overexpression of *IL6* in stage III compared to stage II (*P*=0.03). Data from 87 samples were used. Boxplots show the median (bar), interquartile range values (Q2 and Q3), and maximum and minimal values. The data were downloaded from UALCAN, from the expression profiles for each gene analyzed. Expression and group status were compared by the platform itself using Student t-test. *P<0.05.

Supplementary Figure S6. Tumor gene expression according to nodal metastasis status. Boxplots showing significant overexpression of mRNA of *CD274* in the N3 category compared to N0 (*P*=0.03) and of *CHI3L1* in N0 compared to N1 (*P*=0.03), N2 compared to N1 (*P*=0.04) and N3 compared to N2 (*P*=0.05). *FN1* mRNA overexpression was significantly associated with N3 compared to N0 (*P*=0.0002), N1 (*P*=0.0009), and N2 (*P*=0.0001). Data from 87 samples were used. Boxplots show the median (bar), interquartile range values (Q2 and Q3), and maximum and minimal values. The data were downloaded from UALCAN, from the expression profiles for each gene analyzed. Expression and group status were compared by the platform itself using Student t-test. *P<0.05; **P<0.01.

Supplementary Figure S7. **Prognostic curve of hub genes.** The prognostic significance of the 18 TAM genes involved in TME signaling pathways from patients with MPM. Kaplan-Meier survival curves show the association between the TAM genes and OS. Red lines indicate high expression (>3rd quartile) and blue lines indicate low/medium expression (≤3rd quartile). Significant correlations were seen between high expression of *NOS2* and poor OS (*P*=0.049), high expression of *INHBA* with poor OS (*P*<0.0001), and high expression of *FN1* and poor OS (*P*<0.0001). Data from 87 samples were used. Kaplan-Meier curves were downloaded from UALCAN, from the expression profiles for each of the genes analyzed. The significance of survival impact was measured by log-rank test, and *P*-values were provided by the UALCAN platform.
