# Supplementary figures and images for: The Immunological Landscape of M1 and M2 Macrophages and Their Spatial Distribution in Patients with Malignant Pleural Mesothelioma
Source: Cancers (Basel). 2023 Oct 24;15(21):5116. doi: 10.3390/cancers15215116 (PMC10650059; doi:10.3390/cancers15215116)

# Supplementary Figure S1

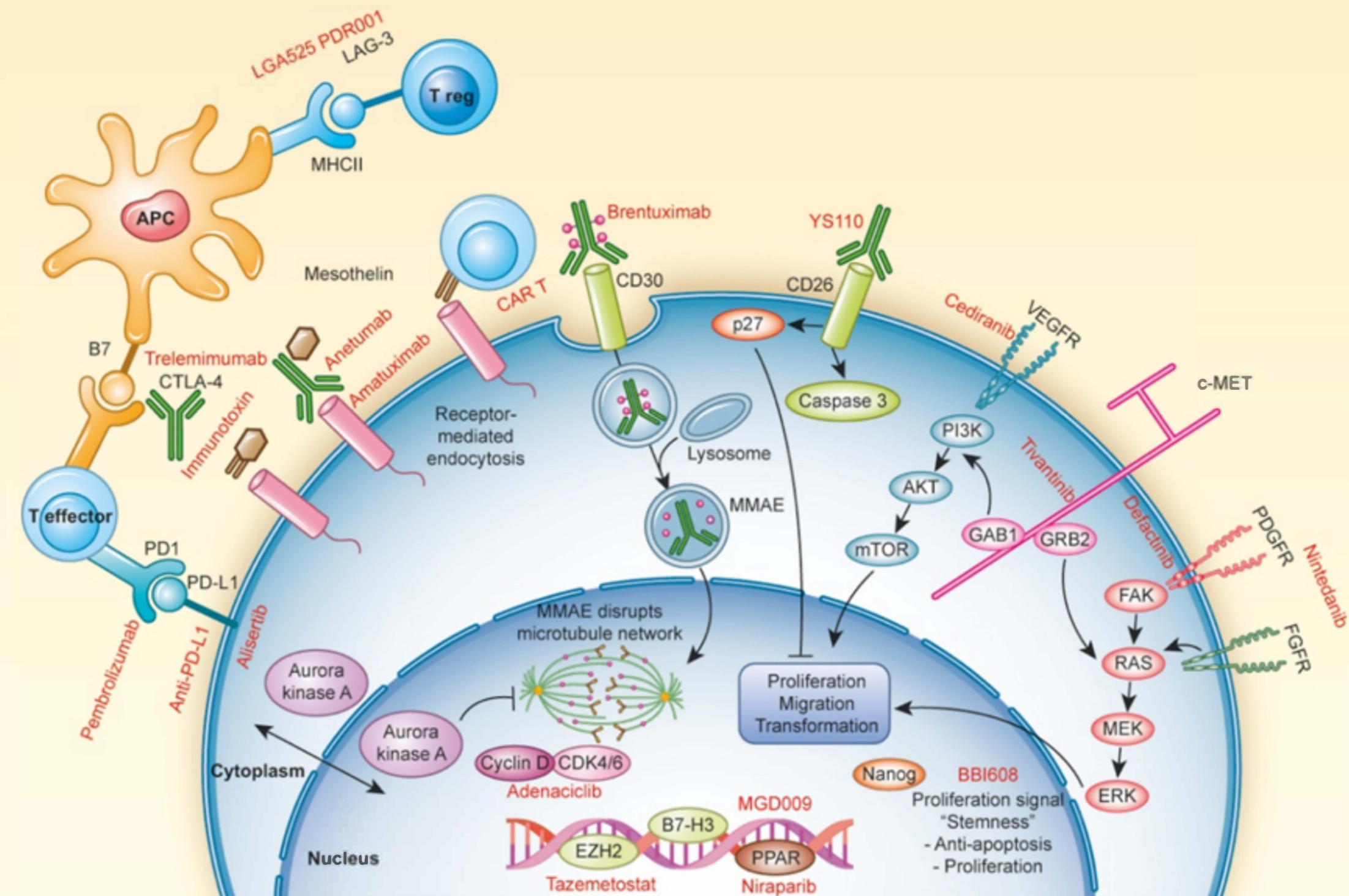

Supplement: Supplementary file 1 [file cancers-15-05116-s001.zip › Supplementary Figure S1.pdf]

Supplementary Figure S2

Reactive Human Tonsil

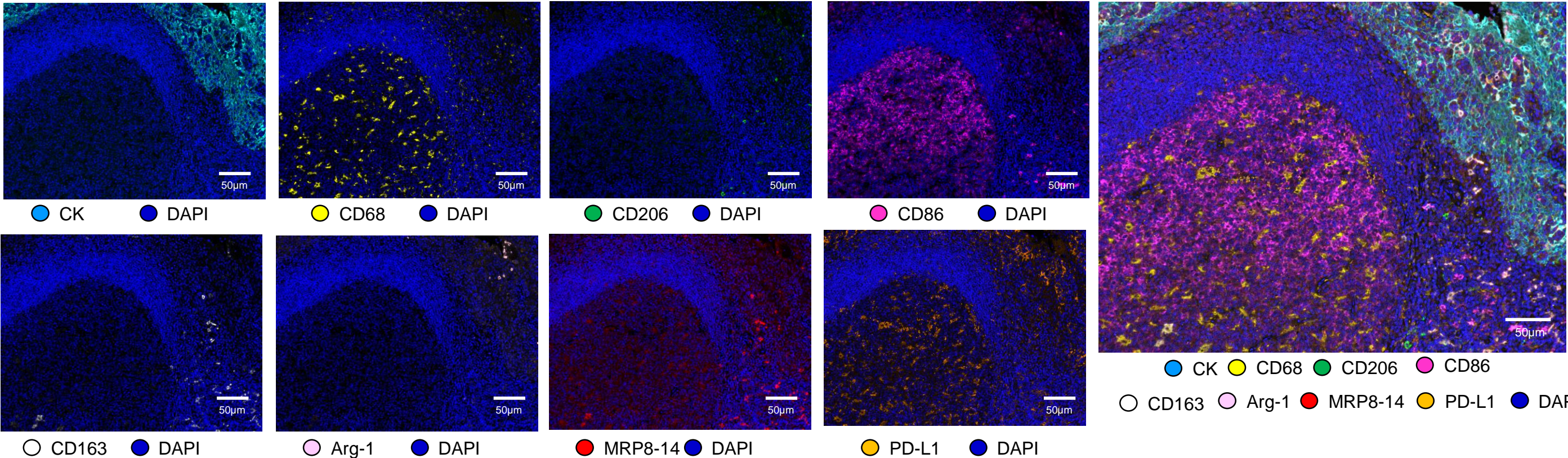

Acute Tuberculous Pneumonia

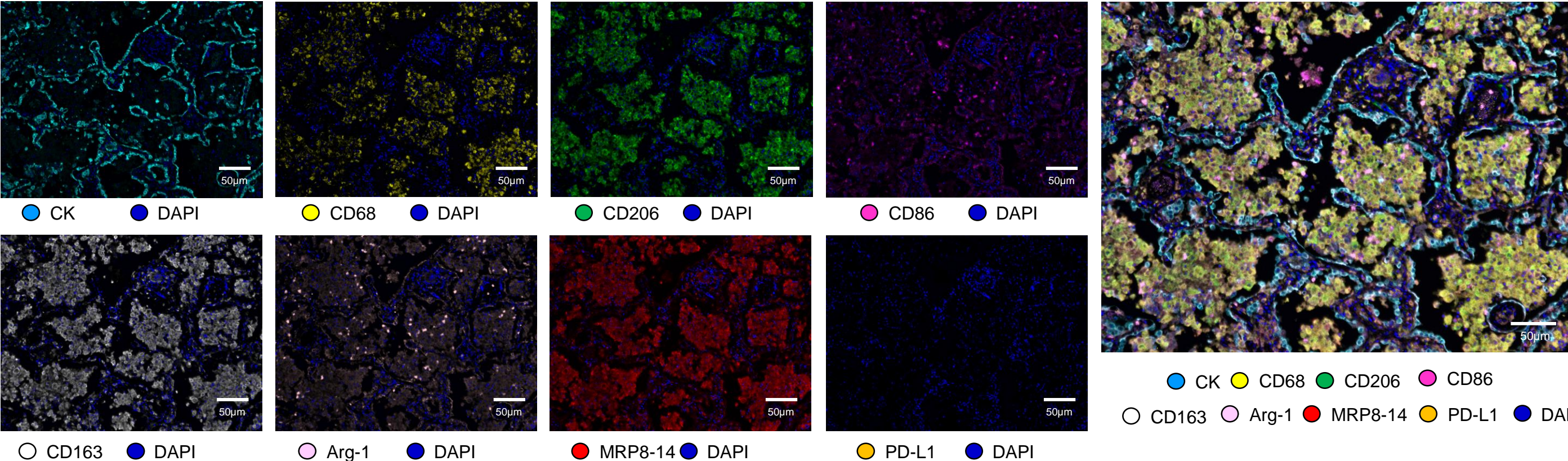

Supplement: Supplementary file 1 [file cancers-15-05116-s001.zip › Supplementary Figure S2.pdf]

**Supplementary Figure S3**

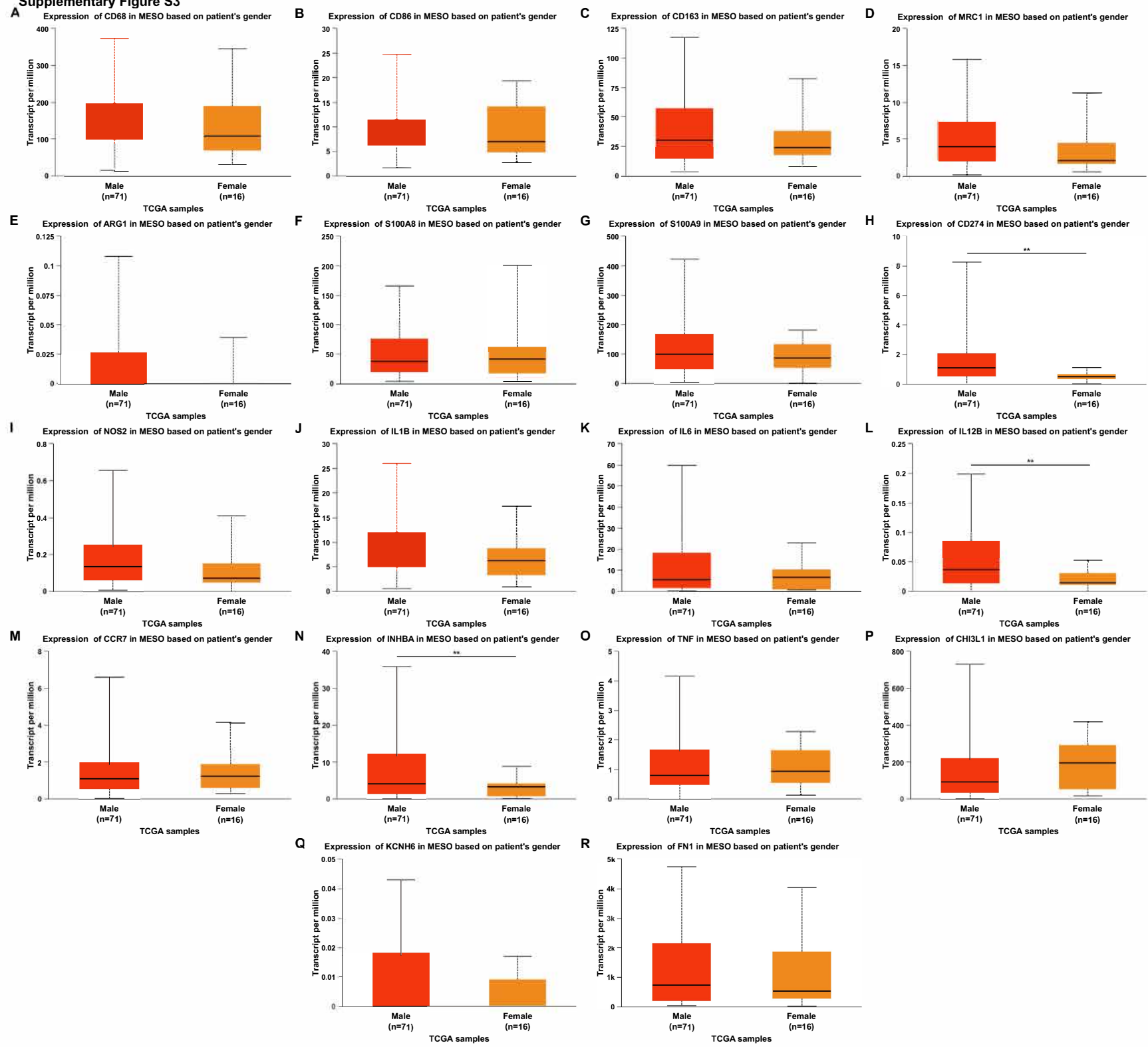

Supplement: Supplementary file 1 [file cancers-15-05116-s001.zip › Supplementary Figure S3.pdf]

Supplementary Figure S4

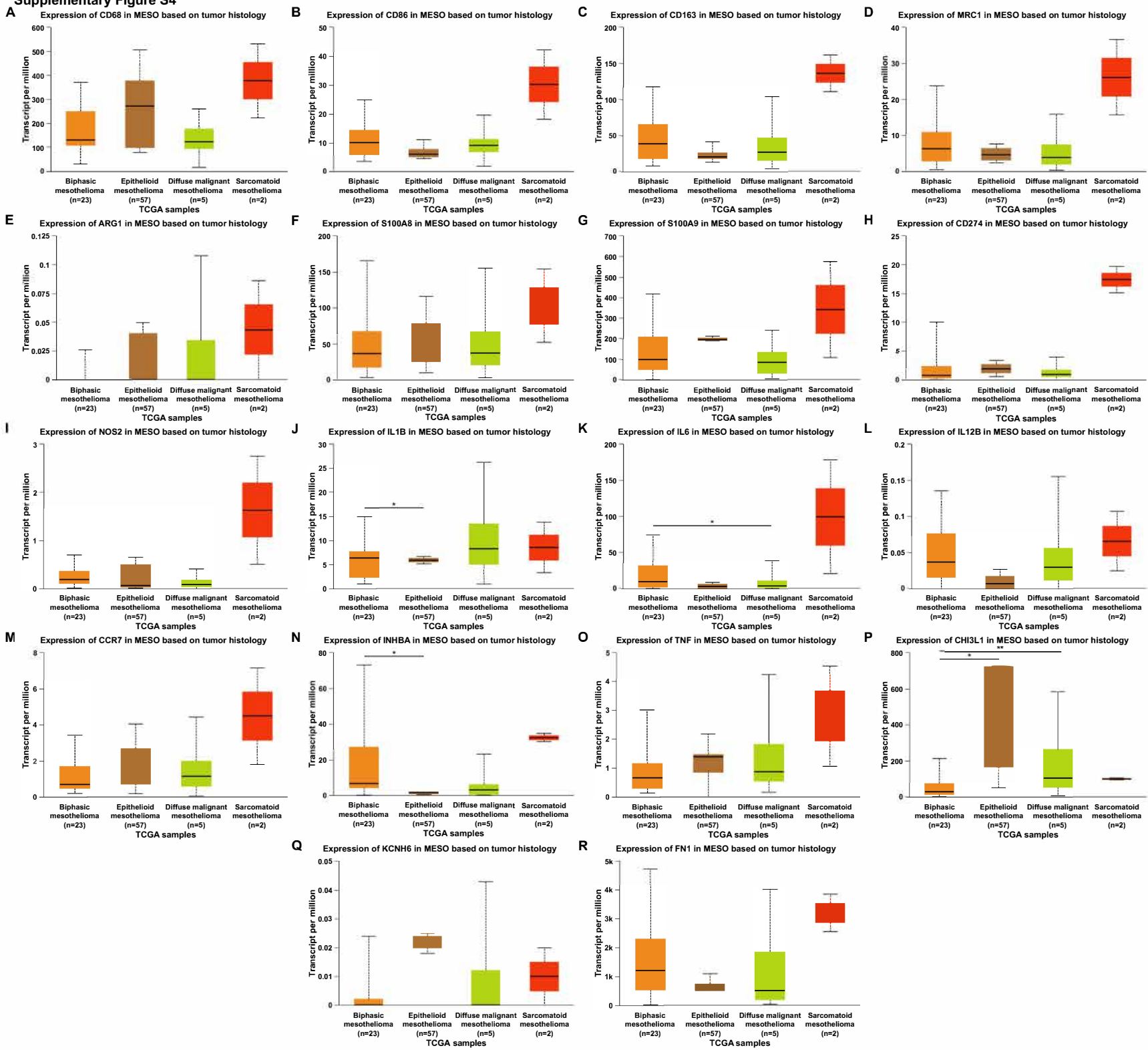

Supplement: Supplementary file 1 [file cancers-15-05116-s001.zip › Supplementary Figure S4.pdf]

**Supplementary Figure S5**

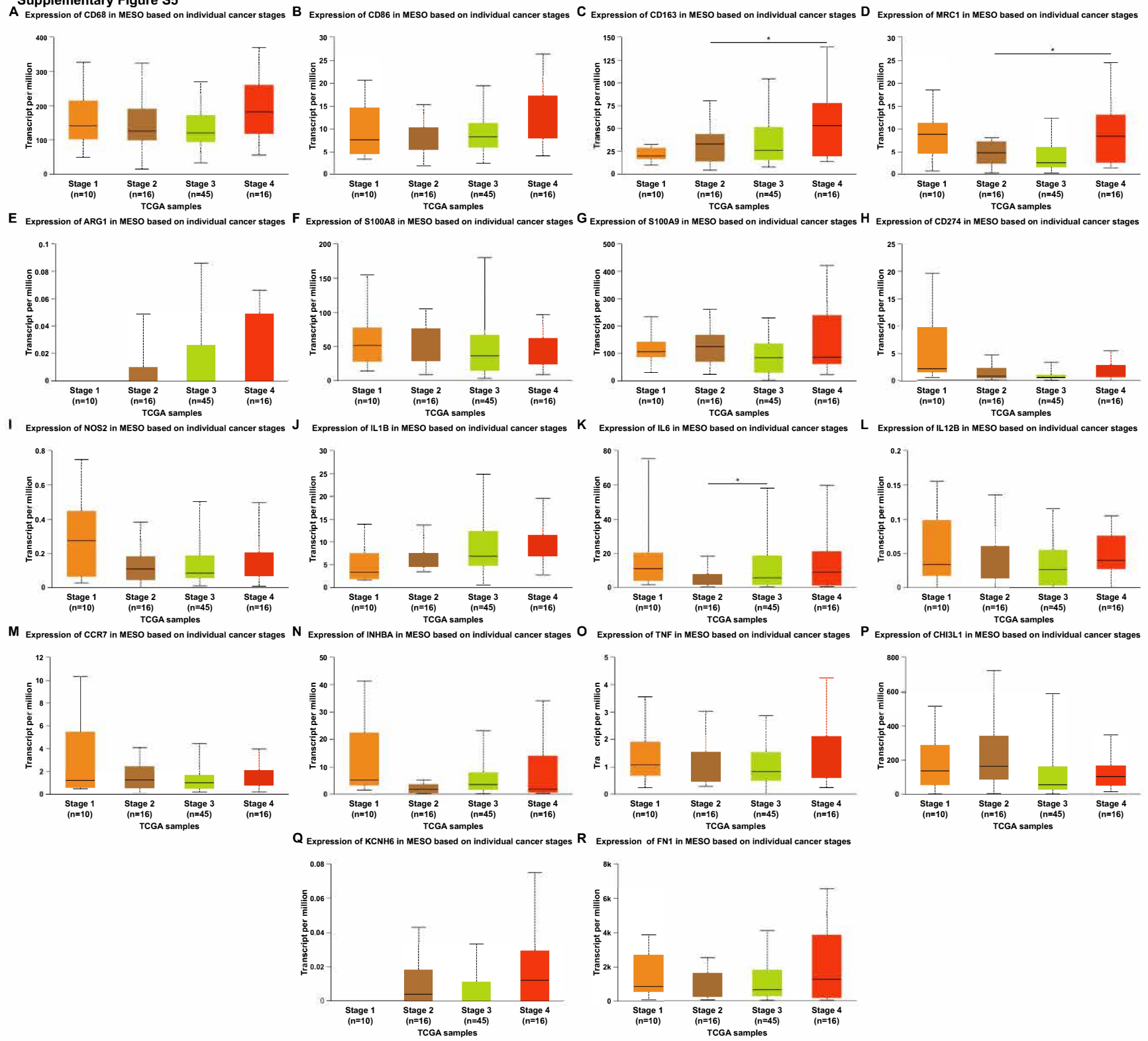

Supplement: Supplementary file 1 [file cancers-15-05116-s001.zip › Supplementary Figure S5.pdf]

**Supplementary Figure S6**

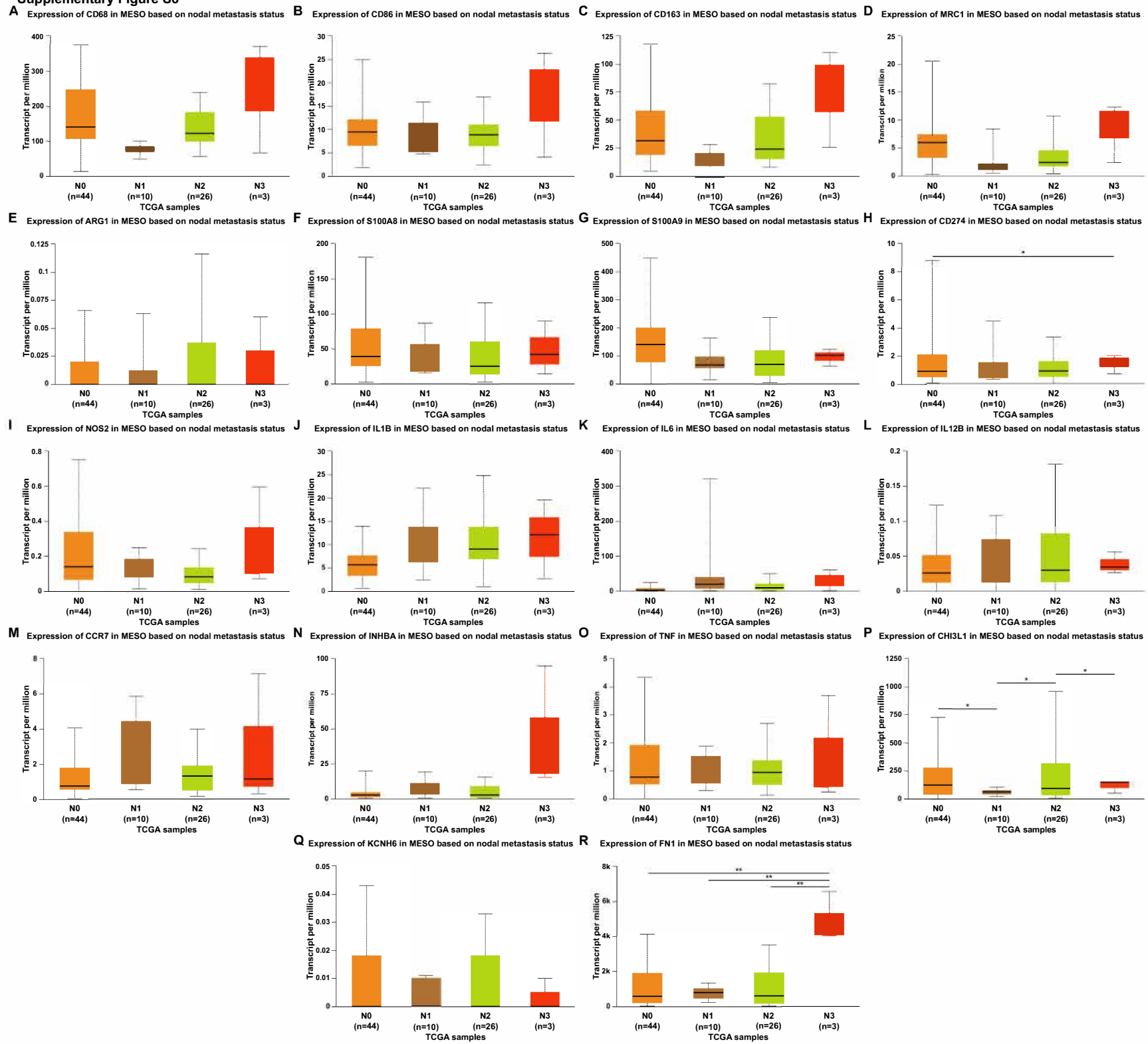

Supplement: Supplementary file 1 [file cancers-15-05116-s001.zip › Supplementary Figure S6.pdf]

**Supplementary Figure S7**

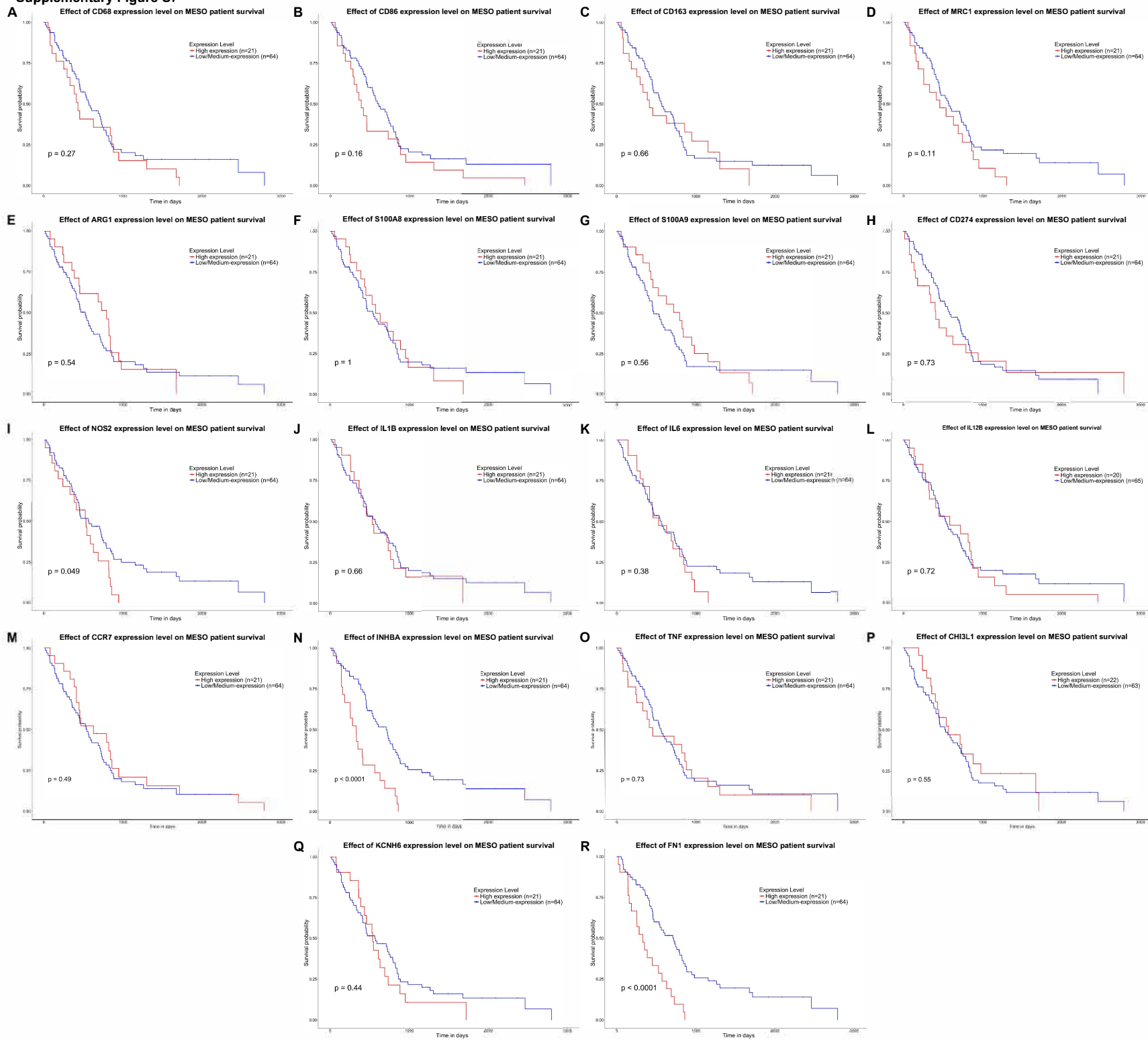

Supplement: Supplementary file 1 [file cancers-15-05116-s001.zip › Supplementary Figure S7.pdf]
